# Fathers’ experiences during their partners’ childbirth and postpartum period: health vulnerabilities during the COVID-19 pandemic

**DOI:** 10.1590/0034-7167-2025-0108

**Published:** 2026-07-06

**Authors:** Maria Adelane Monteiro Silva, Antonia Tainá Bezerra Castro, Francisco Wellington Dourado, Jessica Ketleen Caetano Lopes, Maria do Socorro Melo Carneiro, Rojelma Carneiro Ferreira

**Affiliations:** I Universidade Estadual Vale do Acaraú. Sobral, Ceará, Brazil; II Universidade Federal do Ceará. Sobral, Ceará, Brazil; III Universidade Estadual do Ceará. Fortaleza, Ceará, Brazil; IV Universidade de Fortaleza. Sobral, Ceará, Brazil

**Keywords:** Health Vulnerability, Pandemics, SARS-CoV-2, Paternity, Labor, Obstetric., Vulnerabilidad en Salud, Pandemias, COVID-19, Paternidad, Trabajo de Parto.

## Abstract

**Objectives:**

to analyze fathers’ experiences during their partners’ childbirth and postpartum period during the COVID-19 pandemic, with an emphasis on health vulnerabilities.

**Methods:**

a qualitative study conducted between August 2020 and July 2021 in two maternity wards located in two hospitals in a municipality in the semi-arid region of Ceará, Brazil. The study included 11 fathers who were with their partners during the childbirth and postpartum period. The thematic analysis technique was used, supported by WebQDA^®^ version 3.0. The Conceptual Model of Health Vulnerability supported the analysis and interpretation of results.

**Results:**

the categories of analysis “Paternal support and the psycho-emotional situation during the COVID-19 pandemic” and “Gender issues, functional literacy and paternal participation” emerged.

**Final Considerations:**

fathers’ experiences during their partners’ childbirth and postpartum period during the COVID-19 pandemic portray situations of health vulnerability. Healthcare services need to be prepared to face future health crises.

## RESUMEN

**Objetivos:**

analizar las experiencias paternas de sus parejas durante el parto y puerperio en el contexto de la pandemia de COVID-19, con énfasis en las vulnerabilidades en salud.

**Métodos:**

estudio cualitativo realizado entre agosto de 2020 y julio de 2021 en dos maternidades de dos hospitales de un municipio de la región semiárida de Ceará. Once padres que acompañaron a sus parejas durante el parto y puerperio participaron en el estudio. Se utilizó el análisis temático, con el apoyo de WebQDA^®^ versión 3.0. El Modelo Conceptual de Vulnerabilidad en Salud fundamentó el análisis e interpretación de los resultados.

**Resultados:**

surgieron las categorías de análisis “Apoyo paterno y situación psicoemocional durante la pandemia de COVID-19” y “Cuestiones de género, alfabetización funcional y participación paterna”.

**Consideraciones Finales:**

las experiencias paternas de sus parejas durante el parto y puerperio en la pandemia de COVID-19 evidencian situaciones de vulnerabilidad en salud. Los servicios de salud deben estar preparados para afrontar futuras crisis sanitarias.

## INTRODUCTION

Childbirth and birth are marked by significant personal, emotional, and sociocultural experiences related to postpartum women’s preferences, family, professionals’ approach, the conduct adopted during care, among other factors. Therefore, in addition to adopting good practices in childbirth and birth care, paternal involvement during the pregnancy and postpartum period is recommended, as this practice can provide women with security and well-being^(1-3)^.

National and international initiatives seek positive birth experiences, emphasizing women’s partners during this time by a person of their choice, as well as providing safe and respectful care. In this regard, Law 11,108 of 2005 regulated, in Brazil, the presence of a companion of the woman’s choice during prenatal care and all phases of hospitalization, including labor, childbirth, and the immediate postpartum period, in Brazilian Health System (In Portuguese, *Sistema Único de Saúde* - SUS) services, whether its own network or affiliated^(4)^. Partner prenatal care, along with the production of a guide for healthcare professionals, is also a strategy adopted in the country^(5)^.

However, despite the increased visibility of this issue worldwide in recent decades, healthcare services have not yet been able to effectively implement strategies that achieve partner involvement, which can negatively impact women’s satisfaction with the childbirth process^(1,4,6)^. Healthcare facilities need to be prepared to welcome fathers/partners so that actions can be strengthened^(5)^.

With the advent of the COVID-19 pandemic, caused by SARS-CoV-2, there was a need to reorganize care flows in order to prioritize measures to control and reduce contagion, without harming pregnant women. It is worth noting that some maternity hospitals adopted isolation during childbirth, given the initial scenario of uncertainty about vertical transmission of the infection^(7,8)^.

In this context, pregnant women experienced feelings of fear and worry due to the suspension of their right to a companion, which weakened their coping mechanisms due to the physical absence of emotional support and family support. Paternal presence constitutes one of the main supports during the childbirth and postpartum period, as it promotes relevant benefits, such as the development of paternal identity and interaction between couples, through fathers’ participation in assisting the progression of their partners’ labor^(9,10)^.

Moreover, the pandemic context has weakened healthcare practices, including maternal and child care, making it impossible, among other strategies, for fathers to be present with women during childbirth, thus placing pregnant women, their partners, and family members in a vulnerable position^(11,12)^. On the other hand, the pandemic also led to the possibility of some parents remaining in home office, which facilitated closer relationships with family. However, it is understood that this closeness needs to be encouraged and nurtured so that vulnerable factors can be minimized^(13)^.

For this study, we used the conceptual model proposed by Florêncio and Moreira, which clarifies the concept of health vulnerability (HV)^(14)^, understood as a condition of human life constructed through power relations between the subject and social elements: subject is constituted from subjective relations; and social considers the scene of appearance where a subject interacts with the other. These elements are attributes of human life and are supported by concepts and subconcepts. In the conceptual model, it is believed that the power relations of the subject-social elements potentiate situations of precariousness when empowerment is not experienced by individuals. Therefore, fostering individuals’ and communities’ empowerment becomes imperative in a context of crisis, such as the COVID-19 pandemic. Concerning paternal involvement during the childbirth and postpartum period, it is essential to seek strategies that allow positive experiences to be lived even in adverse social settings, contributing to the reduction of vulnerabilities.

From this perspective, the following questions emerged: what are the fathers’ experiences during the childbirth and postpartum period during the COVID-19 pandemic? What vulnerabilities are present in this context? Therefore, it becomes opportune to study the experience of fathers’ participation in times marked by COVID, with a view to formulating new strategies for addressing these vulnerabilities also in the post-pandemic scenario, since studies addressing this topic are still incipient in the scientific literature.

It is important to highlight the possibility of new pandemics arising from emerging and re-emerging diseases in the public health field. Although the COVID-19 pandemic was controlled in 2022, it is believed that understanding the fathers’ role during the pregnancy and postpartum period in this scenario will contribute to improving healthcare practices to humanize and enhance care for women, as well as planning actions that meet fathers’ and families’ needs in adverse situations. It is worth noting that in Brazil, the pandemic manifested itself as not only a health crisis, but also a social and political one, affecting different social groups. Therefore, it is still necessary to reflect on and discuss the recent experience of this period and its repercussions^(15)^.

## OBJECTIVES

To analyze fathers’ experiences during their partners’ childbirth and postpartum period during the COVID-19 pandemic, with an emphasis on HVs.

## METHODS

### Ethical aspects

The study was approved by the *Universidade Estadual Vale do Acaraú* Research Ethics Committee, following Resolution 466 of the Brazilian National Health Council. In order to guarantee participant confidentiality and anonymity, the letter “P” followed by an Arabic numeral was used, according to the order of the interviews.

### Study design, period, and location

This qualitative study was conducted between August 2020 and July 2021 in two maternity wards located in two hospitals in a municipality in the semi-arid region of Ceará, Brazil. One hospital is a philanthropic institution, a regional obstetric reference center, and serves patients affiliated with the SUS. The other is a private hospital, serving both private and supplementary healthcare providers. The study locations were intentionally chosen to identify the diverse unique experiences of subjects as comprehensively as possible. The COnsolidated criteria for REporting Qualitative research checklist was used to ensure study rigor and quality^(16)^.

### Sample; inclusion and exclusion criteria

Eleven fathers who were with their partners during the childbirth and postpartum period participated in the study. Fathers who stayed with their partners during labor, regardless of the outcome of childbirth, were included. Fathers who were only briefly present at the maternity ward with their partner were excluded. Due to the homogeneous nature of the sample, this was defined through theoretical saturation, identified from the redundancy and repetition of information in the discourse^(17)^.

### Data collection

Data collection took place between January and March 2021, through individual interviews using a semi-structured script containing questions about identification and characterization of fathers and about their experiences during the childbirth and postpartum period with their partner. The interviews were recorded after obtaining authorization from participants, through the signing of an Informed Consent Form, at which time the researcher, one of the authors of this study, introduced herself as a nurse and a student in the graduate program at the *Universidade Federal do Ceará*, explaining the research objectives and seeking to make them feel comfortable not to participate.

Participants were randomly selected from daily visits to hospital maternity wards and approached individually. Interviews were conducted according to fathers’ availability and the service’s dynamics, taking place in a private location preferred by the interviewees. Therefore, they occurred in hospital waiting rooms and rooming-in wards, adhering to current sanitary measures to prevent viral spread. Interviews lasted 30 to 60 minutes, with questions moderated by the previously trained lead researcher.

### Data analysis

After recording the interviews, the information obtained was transcribed in full and then revised for spelling, without altering the essence. The thematic analysis technique, related to the content analysis method, was employed, which has three chronological phases: pre-analysis; analysis; treatment of results, and interpretation^(18)^. This method is suitable for the object of this study since it seeks to substantially understand the subjective issues involved in experiences during the childbirth and postpartum period during the pandemic.

In the preliminary analysis, a cursory reading of interviews was conducted to formulate hypotheses, highlighting words, text fragments, and phrases. Following this, the content was explored through message coding, aiming to identify previously defined core meanings, which were then grouped to create thematic categories. After categorization, the data obtained were processed and interpreted^(16)^ based on the Conceptual Model of Health Vulnerability^(14)^. The model is organized around its two main elements: the individual and the social. The individual element involves concepts and sub-concepts that reflect the physical and psycho-emotional situations, and individuals’ educational attainment, functional literacy, behavior, and interpersonal relationships. The social element encompasses sub-concepts ranging from socioeconomic and income conditions to environmental, programmatic, and political contexts. These elements are neither hierarchical nor dissociated, but are multiple and interconnected, and in continuous motion, producing situations of HV.

WebQDA^®^ version 3.0^(19-21)^ also supported qualitative data analysis. The use of this software aided in organizing the initial categories, supporting the decision on the inclusion of certain thematic categories, as well as indicating possibilities for excluding those that were less relevant. In this way, it improved the process of categorizing and interpreting the data.

## RESULTS

Eleven men were interviewed, of whom three were between 24 and 26 years old, four between 28 and 31 years old, and four between 34 and 39 years old. All declared themselves to be brown, and regarding education, four had completed high school, three completed higher education, two completed elementary school, one incomplete high school education, and one incomplete elementary school education. Regarding occupation, a variety of professions were observed, with two being farmers.

Six reported being in a stable union, and five were married. Concerning family income, three had no fixed income, three had one to two wages, two had two to three wages, and three had more than three wages. Only four resided in the municipality where the hospitals are located. Thus, based on these characteristics, distinct realities were observed among the fathers assisted in healthcare services, especially from the perspective of education and family income, where the private hospital users have a more favorable socioeconomic situation than those of the philanthropic hospital. With regard to previous paternal experience, only three interviewees were already fathers, all of whom were interviewed at the philanthropic hospital.

The following categories of analysis emerged: Paternal support and the psycho-emotional situation during the COVID-19 pandemic; Gender issues, functional literacy, and paternal participation.

### Paternal support and the psycho-emotional situation during the COVID-19 pandemic

Fathers’ statements reveal distinct experiences regarding their participation in the birth of their children. Some were not present for the cesarean section due to hospital protocols related to the COVID-19 pandemic. In contrast, for others, the restrictive measures did not hinder the follow-up process, as observed in P2, P7, P8, and P9:

*I did not participate in the surgery because I was not allowed into the operating room due to the pandemic.* (P2)

*Regarding the pandemic, we did not encounter any obstacles in this process.* (P7)

*We didn’t have any obstacles, they just gave some instructions about the surgical attire and to maintain distance during* [...] (P8)

*So, what we needed was not hindered; I was able to observe.* (P9)

Participants’ statements clarify that the participation of most parents was not affected by the viral containment protocols. It is noteworthy that both hospitals adopted measures to organize services, such as allowing a companion to be present during childbirth. In the philanthropic service, the presence of a companion was only permitted during vaginal delivery. In the private network, there were no restrictions.

Despite the precautions to prevent the spread of the virus, feelings of fear, worry, and insecurity arose among parents regarding the possibility of transmitting the infection to their partner and child, leading to potential negative effects on their participation:

*Certainly, the pandemic has made the process more stressful, including the postpartum period. The newborn requires special care.* (P4)

*In fact, I could say that the current context is a negative point, in the sense that it makes the whole situation more tense and brings more fear.* (P4)

*But the pandemic was, and has been, a complicating factor in our feeling of being at ease during this moment that is so sublime for human beings.* (P4)

*This pandemic has further distanced visitors from the child, and we are afraid of the child getting sick because, if the child gets sick, who will take care of the child?* (P8)

We recognize that the fathers wished to participate in this significant period of their lives. However, the context of the pandemic affected their participation, as they expressed feelings related to accompanying the childbirth and postpartum period, from the perspective that the woman and child are part of a risk group in the pandemic scenario. The interviewees’ statements revealed an awareness of the repercussions of the pandemic on fatherhood.

### Gender issues, functional literacy, and paternal involvement

Gender issues are present in this category, stemming from initiatives that represent a break from the tradition of maternal care, in which the mother is solely responsible for childcare. The statements explicitly demonstrate active parental involvement and a willingness to learn how to care for children. However, it is noted that there is still paternal insecurity in assisting with childcare, as seen in P6, P7, and P8:

*I haven’t held the baby yet.* (P7)

*You have to be careful. I’m afraid of hurting him, of twisting the baby’s spine.* (P8)

*But it was very good; I was able to follow the whole process, see his measurements, the first doses of vaccines. I didn’t know how to hold a baby, so I’m still learning. Since it’s my first child, I’ve already changed his clothes.* (P6)

[...] *I’ve already changed the baby’s diaper, I’ve already cleaned up poop, I learned now. And I rocked the baby all night and tried to get the baby to breastfeed.* (P9)

*I kept changing her when she needed it, cleaning her bottom.* (P11)

Knowledge about the use of non-pharmacological methods to promote pain relief and assist in the birthing process encouraged fathers’ participation in woman care. During the postpartum period, care related to assistance with bathing was reported, as well as partners’ participation and encouragement in breastfeeding.

*Taking her to the bathroom, helping her with the bath* [...]. (P1)

*I put her on the birthing ball, on the birthing stool. I massaged her with my hands, with that birthing stool, I walked with her.* (P2)

*I helped her by putting her on the birthing stool, on the ball, on the bar and I massaged her.* (P5)

*I helped her put the baby to breastfeed, for now.* (P4)

It was also observed that some fathers are unaware of the care that can be offered to the expectant mother, highlighting the need to include partners in prenatal care:

*I don’t know what it is* [...] *they didn’t tell me about these methods.* (P3)

## DISCUSSION

Labor, childbirth, and the immediate postpartum period constitute not only a physiological process but a chain of events marked by emotional, affective, and sociocultural experiences that must be considered in healthcare. This study allowed for a focus on paternal participation during the childbirth and postpartum period during the COVID-19 pandemic, considering that the partner’s presence is a significant factor in providing emotional support and assistance in the care provided to women, giving them security, tranquility, and comfort, which can positively impact women’s reproductive experience^(9)^.

The health crisis arising from the COVID-19 pandemic impacted the weakening of obstetric services due to the need to contain viral transmission. Emergency measures related to the contingency plan being implemented in the country were incorporated into practices for childbirth and immediate postpartum care. However, this occurred at the expense of good obstetric practices with regard to equal access to quality perinatal care and the protection of rights provided for in current legislation^(17)^. Thus, the pandemic context led to situations of vulnerability for the population in both individual and collective dimensions, aggravating and revealing inequalities^(22,23)^. Access to healthcare services stands out as a fundamental second-generation right, and the absence or insufficiency of access to diagnosis, treatment, and supplies constitutes a significant problem. Other aspects such as geographical distance, waiting lists, lack of beds, among others, also contribute to processes of vulnerability^(13)^.

In addition, the restrictive measures imposed by most healthcare institutions were responsible for the temporary change in some practices, such as: prohibition or control of companion(s) during labor, childbirth, and postpartum; absolute restriction of visitors; recourse to unnecessary obstetric interventions; criteria for skin-to-skin contact; umbilical cord clamping and rooming-in; and breastfeeding support^(10,24)^.

In the interviews, it is possible to perceive that the restrictive measures, due to viral containment, prevented some individuals from participating more effectively during the childbirth and postpartum period. This reality is also similar to the international scenario, where it was evident that partners and support persons for women were negatively impacted by restrictions in maternity services during the pandemic, experiencing feelings of isolation, psychological distress, and reduced bonding time with their babies^(25)^. This situation warrants attention, given that fathers’ involvement at this time is relevant, as it has positive effects on the progress of labor, postpartum recovery, and as a source of emotional support^(26)^. Even understanding the importance and necessity of these measures, it is understood that the impacts generated by factors that create vulnerabilities can be mitigated by practices that guarantee humanized care, even in times of health crisis such as that caused by COVID-19^(27)^.

This study observed that restrictive measures in maternity wards during the pandemic period were not implemented uniformly, but were relaxed according to the policy of each institution so that some hospitals maintained, albeit partially, the implementation of the Companion Law^(4)^ in the maternity wards studied. The law was implemented at the national level, about 19 years ago, and yet it is observed that women are unable to fully enjoy the rights guaranteed by the current Law, which constitutes an act of violation of the couple’s reproductive rights by the Women’s Healthcare Services^(28)^, generating a vulnerable condition for the father, his partner and the newborn.

Therefore, the presence of a companion during labor, childbirth, and the postpartum period is considered fundamental, including trustworthy individuals and qualified professionals. It is necessary to recognize the companion as an important social actor at this time, since they reflect on the physiological process of childbirth and strengthen bonds^(2)^.

It was observed that the statements reflect a diversity of feelings and emotions, both positive and negative, surrounding the fathers’ presence during the childbirth and postpartum period. They felt happy to be present, but were also affected by feelings of insecurity and fear of virus transmission. Thus, it is understood that pregnancy is marked by a period of emotional instability, and the pandemic, in general, triggered levels of depression and anxiety in pregnant women, indicating a greater need for family support and improved care provided by healthcare professionals.

Hence, the interviews highlighted the impact on fathers’ psycho-emotional status, including the fear and concern of contaminating his partner and child, triggering negative repercussions on fathers’ effective participation and making them vulnerable. According to the HV model, the psycho-emotional status refers to a condition of subjective experience in a specific context that involves beliefs, feelings, well-being, perceptions, desires, values, and mental health^(14)^.

The study found similar results, identifying fathers’ concern related to possible contagion within the family unit and, therefore, the emergence of a duality of feelings: while, on the one hand, there is the joy of a child’s arrival amidst a chaotic context, on the other hand, the understanding of the existence of the risk of transmission associated with the partner’s presence alongside the woman gives rise to a feeling of guilt and tension^(29)^. This information paints a disturbing picture, as the fear of contracting COVID-19 exacerbates symptoms of depression and anxiety, and creates conflict between spouses^(24,30)^.

Supporting these findings, it was found that the pandemic can cause some mental disorders in men, including paternal postpartum depression^(31)^. Therefore, health authorities’ and professionals’ participation is important in addressing the needs of this group, through the development of actions that encompass screening, diagnosis, prevention, and treatment. It is also emphasized that depression in mothers has been studied during the pandemic, with scientific literature showing that women have been more susceptible to mental disorders. However, fathers’ mental health is still underestimated^(32)^.

Based on this, it is observed that the COVID-19 pandemic imposed conditions of HV on fathers, making it urgent to develop a public healthcare model that encompasses promotion, prevention, and intervention actions within the family, within territories or communities, with the aim of promoting greater paternal participation in adverse contexts. Healthcare professionals should encourage the creation of support spaces for families in vulnerable situations, guaranteeing autonomy, establishing bonds of trust and training agents of change to improve care^(33)^, and better cope with the challenges in times of health crisis^(27)^.

Furthermore, fathers’ statements during the immediate postpartum period relate to their performance in caring for their spouse and child, contributing to the strengthening of bonds and the effective construction of their paternal figure. However, the results revealed fathers who assisted with caregiving demands, but also fathers who expressed insecurity and faced challenges in this reality.

Fathers’ lack of involvement in care practices during the birth and postpartum period may be associated with deficient functional literacy by fathers, which prevents them from seeking and using acquired knowledge in providing healthcare for themselves or for pregnant women and babies^(34)^. Therefore, this situation constitutes a precarious condition that increases the vulnerability of these participants and hinders effective parental participation. It is agreed that fathers without satisfactory literacy skills^(35)^ have their capacity for making health decisions affected. Consequently, their autonomy in the face of care demands is also compromised.

Furthermore, it is understood that cultural issues, stemming from patriarchy and machismo, which reproduce gender inequalities, are still reinforced in society today, especially in regions of the northeast of the country, such as the Brazilian semi-arid region^(36)^, also contributing to this scenario of absence of “fathering”. Therefore, paradigms regarding responsibility for childcare persist, leading to paternal disinvolvement from pregnancy onwards. Gender differences are reflected in the distribution of responsibilities, with the mother taking over the caring and the protecting, while the father becomes responsible for providing materials and resources for the family^(37)^. It is noteworthy that gender inequalities, sexism, and the performance of traditional roles are identified as factors that exacerbate sexual violence. Thus, the childbirth and postpartum period continues to be marked by the presence of a father who is a spectator rather than an active participant.

For this reason, the lack of knowledge and information about non-pharmacological methods of pain relief during childbirth reported by P3 stands out, which illustrates the fragility of the health education offered to these pregnant women’s partners, negatively affecting their involvement during the childbirth process. This situation reflects the programmatic aspect of HV that permeates this context, characterized by the disarticulation of the actors in healthcare services during prenatal care^(15)^. In this study, the programmatic situation is understood to include elements of infrastructure and work processes, as well as situations involving difficulties in access and violations of human rights^(38)^.

Given the presence of these programmatic issues in maternal and child healthcare, HV is intensified, and the capacity for agency among participants in the face of these precarious conditions tends to be compromised. Therefore, prenatal care should be improved and implemented with an emphasis on women’s health needs, strengthening the culture of having a support person present from the initial consultations at the primary care level, in order to provide information about childbirth and postpartum care and contribute to mitigating these HVs^(39)^.

In the meantime, it is fundamental to effectively implement paternal health education to encourage the father’s participation from the pregnancy period to the postpartum period, not only from the perspective of material provision, but above all, in emotional involvement with the pregnant woman, with the baby, and in encouraging the parenting relationship^(19)^. It is believed that fathers who are aware of their role during the childbirth and postpartum period tend to manage their HVs to take effective participation, even in times of crisis.

Experiencing childbirth, not as a spectator but as a direct participant, redefines masculine identity through the reconstruction of the ideal of fatherhood. It is believed, therefore, that the exclusion of the partner from maternity care during the pandemic had negative impacts on the family bond^(39-41)^.

Therefore, it is understood that, starting from the premise that HVs emerge from the power relations between the individual and society, the relational aspect of paternal participation in the childbirth and postpartum period needs to be considered. It is not enough to simply emphasize the father’s physical presence to comply with the Companion Law; rather, in prenatal consultations, the father’s participation and involvement with the mother and baby should be addressed in a relational and dynamic way so that precarious conditions can be managed and vulnerabilities mitigated^(13)^.

Conversely, by treating the father as a static being who merely observes the process, vulnerabilities are intensified, as they cannot be transformed by a subject who does not have a performative identity, capable of re-signifying experiences, discourses, lived experiences, and social practices.

### Study limitations

Failure to include other actors involved in the parturition and postpartum period, which would broaden the perspective on the object of study and the results during the pandemic period, may not reflect experiences in non-pandemic times, which may limit the generalizability of results.

### Contributions to nursing

The results contribute to reflections on HVs involving the paternal experience during the peripartum and postpartum period of their partner, which were intensified during the COVID-19 pandemic. It is observed that the nurses’ role in providing prenatal care to the partner contributes to participants’ empowerment, aiming at addressing their vulnerabilities through effective health education and qualified support. Furthermore, considering the strong influence of nursing in this scenario, the data from this study also point to the need to promote interventions that favor the inclusion of the father figure in the routine care of the partner and newborn, even in non-pandemic times or other possible pandemic contexts.

## FINAL CONSIDERATIONS

The study revealed that paternal experiences during the childbirth and postpartum period were marked by participation that, although not widely restricted during the COVID-19 pandemic, still faces several challenges to be fully realized. Despite the data for this research being collected in the context of the pandemic, it points to a timeless problem: paternal involvement in maternal and child care demands. Vulnerabilities associated with this specific context need to be recognized and mitigated.

Health education focused on parenthood needs to be implemented during prenatal consultations, given the importance of preparing the pregnant woman and her partner to experience the childbirth and postpartum period safely and effectively. It is necessary to reorient primary care services in the post-pandemic context, preparing them, including, to face future crises. To this end, the development of humanized obstetric care is urgent, directed not only towards the appropriate outcome of the birth, but also towards the relational processes that permeate this context and influence positive experiences with regard to paternal involvement from the pregnancy period, to the experience of labor and childbirth, not as a spectator, but as a leading actor in the exercise of his fatherhood.

Further research is suggested comparing the realities between public and private services, as well as studies that focus on assessing the effects of paternal involvement on obstetric outcomes, since the objective of this research is not to exhaust the understanding of the topic, but to open up new possibilities for further studies.

## Data Availability

The research data are available within the article.
