# Supplementary material for: Fathers’ experiences during their partners’ childbirth and postpartum period: health vulnerabilities during the COVID-19 pandemic
Source: Rev Bras Enferm. 2026 Jul 6;79:e20250108. doi: 10.1590/0034-7167-2025-0108 (PMC13336230; doi:10.1590/0034-7167-2025-0108)
Supplement: Translation

# Translation: Experiências paternas no período parturitivo-puerperal de suas companheiras: vulnerabilidades em saúde na pandemia de COVID-19

**DOI:** 10.1590/0034-7167-2025-0108pt

**Authors:** Maria Adelane Monteiro Silva, Antonia Tainá Bezerra Castro, Francisco Wellington Dourado, Jessica Ketleen Caetano Lopes, Maria do Socorro Melo Carneiro, Rojelma Carneiro Ferreira

**Affiliations:** I Universidade Estadual Vale do Acaraú. Sobral, Ceará, Brasil; II Universidade Federal do Ceará. Sobral, Ceará, Brasil; III Universidade Estadual do Ceará. Fortaleza, Ceará, Brasil; IV Universidade de Fortaleza. Sobral, Ceará, Brasil

**Keywords:** Vulnerabilidade em Saúde, Pandemias, COVID-19, Paternidade, Trabalho de Parto.

## Abstract

**Objetivos:**

analisar as experiências paternas durante o período parturitivo-puerperal de suas companheiras em tempos de pandemia de COVID-19, com ênfase nas vulnerabilidades em saúde.

**Métodos:**

estudo qualitativo, realizado entre agosto de 2020 e julho de 2021, em duas maternidades situadas em dois hospitais de um município do semiárido cearense. Participaram da pesquisa 11 pais que estiveram com suas companheiras durante o período parturitivo-puerperal. Empregou-se a técnica de análise temática, subsidiada pelo *software* WebQDA^®^, versão 3.0. O Modelo Conceitual de Vulnerabilidade em Saúde apoiou a análise e interpretação dos resultados.

**Resultados:**

emergiram as categorias de análise “Acompanhamento paterno e a situação psicoemocional durante a pandemia de COVID-19” e “Questões de gênero, letramento funcional e participação paterna”.

**Considerações Finais:**

as experiências paternas no período parturitivo-puerperal de suas companheiras na pandemia de COVID-19 retratam situações de vulnerabilidade em saúde. Os serviços de saúde precisam estar preparados para o enfrentamento de futuras crises sanitárias.

## INTRODUÇÃO

O parto e o nascimento são marcados por experiências significativas tanto pessoais e emocionais quanto socioculturais, que se relacionam com as preferências da parturiente, o familiar, a postura dos profissionais, as condutas adotadas durante o atendimento, entre outros fatores. Desta forma, além da adoção de boas práticas de atenção ao parto e ao nascimento, recomenda-se o envolvimento paterno durante o período gravídico-puerperal, tendo em vista que essa prática é capaz de proporcionar à mulher segurança e bem-estar^(1-3)^.

Iniciativas nacionais e internacionais buscam experiências de parto positivas, com ênfase no acompanhamento da mulher durante esse momento por pessoa de sua escolha, além de contemplar uma atenção segura e respeitosa. Nesse sentido, a Lei n° 11.108 de 2005 regulamentou, no Brasil, a presença, junto à mulher, de um acompanhante de sua escolha durante o pré-natal e todas as fases de internação, incluindo os períodos do trabalho de parto, parto e pós-parto imediato nos serviços do Sistema Único de Saúde (SUS), da rede própria ou conveniada^(4)^. Utilizar o pré-natal do parceiro, com a produção do guia para profissionais de saúde, também constitui estratégia adotada no país^(5)^.

Entretanto, apesar do aumento da visibilidade desta temática nas últimas décadas em todo o mundo, os serviços de saúde ainda não conseguiram implementar, de modo efetivo, estratégias que alcancem o envolvimento do parceiro, o que pode refletir negativamente na satisfação das mulheres em relação ao processo parturitivo^(1,4,6)^. As unidades de saúde precisam estar preparadas para o acolhimento do pai/parceiro para que as ações sejam fortalecidas^(5)^.

Com o advento da pandemia de COVID-19, causada pelo SARS-CoV-2, houve a necessidade de reorganização dos fluxos de atendimentos, de modo a priorizar as medidas de controle e diminuir o contágio, sem prejuízo às gestantes. Cabe destacar que algumas maternidades adotaram o isolamento no momento do parto, diante do cenário inicial de incertezas sobre a transmissão vertical da infecção^(7,8)^.

Nesse contexto, as gestantes vivenciaram sentimentos de medo e preocupação, devido à suspensão do direito ao acompanhante, o que fragilizou os mecanismos de enfrentamento e superação da mulher pela ausência física do apoio emocional e suporte familiar. A presença paterna constitui um dos principais suportes no período parturitivo-puerperal, na medida em que promove benefícios pertinentes, como é o caso do desenvolvimento da identidade paterna e a interação entre os casais, através da participação dos pais no auxílio à progressão do trabalho de parto de suas companheiras^(9,10)^.

Somado a isso, o contexto pandêmico acarretou a fragilização das práticas assistenciais à saúde, inclusive na atenção materno-infantil, inviabilizando, entre outras estratégias, a presença paterna junto à mulher no processo parturitivo, de modo a introduzir a gestante, seu parceiro e familiares em uma condição de vulnerabilidade^(11,12)^. Por outro lado, a pandemia também levou à possibilidade de alguns pais permanecerem em *home office*, o que facilitou a aproximação com a família. Entretanto, entende-se que essa aproximação precisa ser estimulada e cuidada, de forma que os processos vulnerabilizantes possam ser minimizados^(13)^.

Para este estudo, utilizou-se o modelo conceitual proposto por Florêncio e Moreira, que clarifica o conceito de vulnerabilidade em saúde (VS)^(14)^, compreendido como uma condição da vida humana construída por meio das relações de poder entre os elementos sujeito-social: o sujeito é constituído a partir das relações subjetivas; e o social considera a cena de aparição onde o sujeito interage com o outro. Esses elementos são atributos da VS, e são sustentados por conceitos e subconceitos. No modelo conceitual, acredita-se que as relações de poder do sujeito-social potencializam as situações de precariedade, quando o empoderamento não é vivenciado pelo indivíduo. Logo, favorecer o empoderamento do indivíduo e comunidades se torna imperioso em contexto de crise, como a pandemia de COVID-19. No que se refere à participação paterna no período parturitivo-puerperal, é mister buscar estratégias para que experiências positivas possam ser vivenciadas mesmo em cenários sociais adversos, contribuindo para a redução das vulnerabilidades.

Nessa perspectiva, emergiram os seguintes questionamentos: quais as experiências paternas durante o período parturitivo-puerperal em tempos de pandemia de COVID-19? Quais as VSs presentes nesse contexto? Desse modo, torna-se oportuno estudar a vivência da participação de pais em tempos marcados pela COVID, com vistas à formulação de novas estratégias para o enfrentamento dessas vulnerabilidades também no cenário pós-pandêmico, já que ainda são incipientes na literatura científica estudos que abordem a temática em questão.

Salienta-se que existe a possibilidade do advento de novas pandemias diante das doenças emergentes e reemergentes no âmbito da saúde pública. Embora a pandemia de COVID-19 tenha sido controlada em 2022, acredita-se que compreender a participação paterna no período gravídico-puerperal nesse cenário subsidiará o aprimoramento das práticas assistenciais nos serviços de saúde para humanização e qualificação do cuidado às mulheres, bem como o planejamento de ações condizentes com as necessidades do pai e família em situações adversas. Ressalta-se que, no Brasil, a pandemia se manifestou como uma crise não apenas sanitária, mas também social e política, que atingiu diferentes grupos sociais, sendo necessário ainda refletir e discutir sobre a experiência recente desse período e suas repercussões^(15)^.

## OBJETIVOS

Analisar as experiências paternas durante o período parturitivo-puerperal de suas companheiras em tempos de pandemia de COVID-19 com ênfase nas VSs.

## MÉTODOS

### Aspectos éticos

O estudo foi aprovado pelo Comitê de Ética em Pesquisa com Seres Humanos da Universidade Estadual Vale do Acaraú, seguindo a Resolução nº 466 do Conselho Nacional de Saúde. Com o intuito de garantir a confidencialidade e anonimato dos participantes, usou-se a letra “P” seguido de um número arábico, de acordo com a ordem das entrevistas.

### Desenho, período e local do estudo

Estudo qualitativo realizado no período de agosto de 2020 a julho de 2021 em duas maternidades situadas em dois hospitais de um município do semiárido cearense. Um dos hospitais é caracterizado como filantrópico, constituindo-se referência obstétrica para região, e é responsável por atender à rede conveniada ao SUS. O outro configura-se como privado, contemplando a rede suplementar e clientela particular. A escolha dos locais do estudo se deu intencionalmente, com o intuito de captar, de maneira mais abrangente possível, as diversas singularidades das experiências do objeto em questão. Adotou-se o *checklist COnsolidated criteria for REporting Qualitative research* para garantir o rigor e a qualidade do estudo^(16)^.

### Amostra; critérios de inclusão e exclusão

Participaram da pesquisa 11 pais que estiveram com suas companheiras durante o período parturitivo-puerperal. Incluíram-se os pais que ficaram com suas companheiras no trabalho de parto, independentemente do desfecho do parto. Excluíram-se os pais que estiveram nas maternidades de forma pontual com sua companheira. Pelo caráter homogêneo da amostra, esta foi definida por meio da saturação teórica, identificada a partir da redundância e repetição de informações nos discursos^(17)^.

### Coleta de dados

A coleta de dados ocorreu entre janeiro e março de 2021, por meio de entrevista individual, com roteiro semiestruturado contendo questões sobre a identificação e caracterização dos pais e sobre suas experiências durante o acompanhamento no período parturitivo-puerperal da companheira. As entrevistas foram gravadas após a autorização dos participantes, mediante assinatura do Termo de Consentimento Livre e Esclarecido, quando a pesquisadora, uma das autoras deste estudo, apresentou-se enquanto enfermeira e estudante do programa de pós-graduação da Universidade Federal do Ceará, expondo os objetivos da pesquisa e procurando deixá-los à vontade para não participação.

Os participantes foram selecionados aleatoriamente, a partir de visitas diárias às maternidades dos hospitais, e abordados individualmente. As entrevistas se deram conforme a disponibilidade do pai e da dinâmica do serviço, sendo aplicadas em local reservado, de preferência dos depoentes. Logo, aconteceram nas salas de espera dos hospitais e nas enfermarias de alojamento conjunto, seguindo adequadamente as medidas sanitárias vigentes para prevenção da propagação viral. Tiveram duração de 30 a 60 minutos, com questionamentos mediados pela pesquisadora responsável devidamente treinada previamente.

### Análise dos dados

Após a gravação das entrevistas, as informações obtidas foram transcritas na íntegra e, em seguida, revisadas em relação à ortografia, sem que a essência fosse modificada. Empregou-se a técnica de análise temática, referente ao método de análise de conteúdo, que possui três polos cronológicos: pré-análise; análise; tratamento dos resultados e interpretação^(18)^. Esse método se adequa ao objeto deste estudo, uma vez que busca compreender substancialmente as questões subjetivas que envolvem as experiências durante o período parturitivo-puerperal no contexto da pandemia.

Na pré-análise, realizou-se a leitura flutuante das entrevistas para formulação de hipóteses, destacando-se palavras, fragmentos textuais e frases. Em seguida, ocorreu a exploração do conteúdo, por meio da codificação das mensagens, visando alcançar os núcleos de sentido identificados previamente que foram agrupados, originando as categorias temáticas. Posteriormente, na categorização, efetuou-se o tratamento e interpretação dos dados obtidos^(16)^, apoiando-se no Modelo Conceitual de Vulnerabilidade em Saúde^(14)^. O modelo se organiza em seus dois elementos principais: sujeito e social. O elemento sujeito envolve conceitos e subconceitos que traduzem as situações física e psicoemocional, de escolaridade, de letramento funcional, de seu comportamento, bem como das relações interpessoais do sujeito. O social abrange subconceitos que remetem desde as condições socioeconômicas e de renda até contextos ambientais, programáticos e políticos. Esses elementos não são hierárquicos e nem dissociados, mas são múltiplos e estão interconectados e em contínuo movimento, produzindo situações de VS.

O *software* WebQDA^®^, versão 3.0^(19-21)^, também subsidiou a análise dos dados qualitativos. O uso do *software* auxiliou na organização das categorias iniciais, apoiando na decisão sobre a inclusão de algumas categorias temáticas, bem como apontando possibilidades de exclusão daquelas menos relevantes. Dessa forma, qualificou o processo de categorização e interpretação dos dados.

## RESULTADOS

Entrevistaram-se 11 homens, dos quais três possuíam faixa etária entre 24 e 26 anos, quatro, entre 28 e 31 anos, e quatro, entre 34 e 39 anos. Todos declararam-se pardos, e quanto à escolaridade, quatro tinham ensino médio completo, três, ensino superior completo, dois, ensino fundamental completo, um, ensino médio incompleto, e um, ensino fundamental incompleto. Com relação à ocupação, observaram-se variadas profissões, sendo que dois eram agricultores.

Seis relataram manter união estável, e cinco eram casados. No que concerne à renda familiar, constataram-se três sem renda fixa, três com um a dois salários, dois com dois a três salários e três com mais de três salários. Apenas quatro residiam no município em que se situam os hospitais. Dessa forma, com base nessas características, perceberam-se distintas realidades entre os pais atendidos nos serviços de saúde, especialmente na perspectiva da escolaridade e renda familiar, em que os usuários do hospital da rede privada dispõem de situação socioeconômica mais favorável do que os do hospital filantrópico. No que diz respeito à experiência paterna anterior, apenas três entrevistados já eram pais, compondo os entrevistados atendidos no hospital filantrópico.

Emergiram as seguintes categorias de análise: Acompanhamento paterno e a situação psicoemocional durante a pandemia de COVID-19; Questões de gênero, letramento funcional e participação paterna.

### Acompanhamento paterno e a situação psicoemocional durante a pandemia de COVID-19

Podem-se observar nos discursos dos pais experiências distintas na participação no nascimento de seus filhos. Alguns não estiveram presentes na cirurgia cesariana devido aos protocolos hospitalares relacionados à pandemia de COVID-19. Em contrapartida, para outros, as medidas restritivas não causaram obstáculo no processo de acompanhamento, como observado nos depoimentos de P2, P7, P8 e P9:

*Não participei da cirurgia, pois não permitiram a entrada no centro cirúrgico devido à pandemia.* (P2)

*Com relação à pandemia, não tivemos nenhum obstáculo nesse processo.* (P7)

*Não tivemos obstáculos, só fizeram algumas orientações sobre a vestimenta da cirurgia e para manter a distância durante* [...]. (P8)

*Assim, o que a gente necessitava não houve obstáculo; eu pude acompanhar.* (P9)

As falas dos participantes elucidam que a maioria dos pais não teve a sua participação afetada diante dos protocolos de contenção viral. Destaca-se que ambos os hospitais adotaram medidas de organização dos serviços, como a presença do acompanhante no parto. No serviço de caráter filantrópico, permitia-se a presença do acompanhante apenas no parto normal. Na rede privada, não havia restrição.

Apesar das precauções para evitar a disseminação do vírus, constata-se o surgimento de sentimentos de medo, preocupação e insegurança dos pais em transmitir a infecção para a companheira e o filho, acarretando possíveis efeitos negativos sobre a participação:

*Com certeza, a pandemia tornou o processo mais tenso, inclusive o pós-parto. O recém-nascido tem que ter um cuidado especial.* (P4)

*Aliás, poderia dizer que o atual contexto é um ponto negativo, no sentido que torna toda a situação mais tensa e traz mais medo.* (P4)

*Mas a pandemia foi, e tem sido, um dificultador no nosso sentimento de estar à vontade nesse momento que é tão sublime do ser humano.* (P4)

*Essa pandemia veio para afastar mais os visitantes da criança, e a gente sente medo da criança e dela pegar porque, se ela pegar, quem vai cuidar da criança?* (P8)

Reconhecemos que os pais desejaram participar desse período tão significante em suas vidas. Contudo, o contexto da pandemia afetou a sua participação, ao demonstrarem sentimentos vinculados ao acompanhamento do período parturitivo-puerperal, partindo do ponto de vista que a mulher e o filho integram grupo de risco no cenário pandêmico. Percebeu-se, na fala dos depoentes, o reconhecimento das repercussões da pandemia para atuação paterna.

### Questões de gênero, letramento funcional e participação paterna

As questões de gênero se apresentam nesta categoria, a partir de iniciativas que representam o rompimento da tradição do cuidado materno, em que a mãe é a única responsável pelos cuidados com a criança. Os depoimentos explicitam a inserção parenteral ativa e a disposição para aprender a cuidar do filho. Contudo, constata-se que ainda há insegurança paterna no auxílio dos cuidados, como visto no relato de P6, P7 e P8:

*Não peguei ainda na criança ainda.* (P7)

*Tem que ter cuidado. Tenho medo de machucar, entortar a coluna da criança.* (P8)

*Mas foi muito bom pude acompanhar todo o processo, ver as medidas dele, as primeiras doses de vacinas. Eu não sabia segurar uma criança, então estou aprendendo ainda. Por ser meu primeiro filho, já fiz a troca de roupinha.* (P6)

[...] *já troquei a fralda da criança, já limpei coco, aprendi agora. E nanei o bebê a madrugada inteira e tentava colocar o bebe para mamar.* (P9)

*Eu fiquei trocando ela nas necessidades dela, limpando o bumbum.* (P11)

O conhecimento sobre a utilização dos métodos não farmacológicos para promover o alívio da dor e auxiliar no processo de nascimento favoreceu a participação dos pais no cuidado à mulher. No puerpério, relataram-se cuidados relacionados ao apoio para o banho, e ainda, a participação e incentivo do companheiro na amamentação.

*Levar ela ao banheiro, ajudar no banho* [...]. (P1)

*Coloquei-a na bola, no cavalinho. Fiz massagem com as mãos, com aquele cavalinho, andei com ela.* (P2)

*Ajudei ela colocando no cavalinho, na bola, na barra e fiz massagem.* (P5)

*Ajudei a colocar o bebê para mamar, por enquanto.* (P4)

Percebeu-se, ainda, que alguns pais desconhecem os cuidados que podem ser oferecidos à parturiente, apontando para a necessidade de inclusão dos parceiros no cuidado pré-natal:

*Não sei o que é* [...] *não me falaram desses métodos.* (P3)

## DISCUSSÃO

O trabalho de parto, parto e pós-parto imediato configuram não apenas um processo fisiológico, mas uma cadeia de eventos marcados por experiências emocionais, afetivas e socioculturais que devem ser consideradas no cuidado em saúde. O estudo permitiu direcionar olhares sobre a participação paterna no período parturitivo-puerperal na pandemia de COVID-19, considerando que a presença do companheiro é um fator significativo para o apoio emocional e para o suporte nos cuidados atribuídos à mulher, concebendo a ela segurança, tranquilidade e conforto, o que pode refletir de forma positiva na experiência reprodutiva das mulheres^(9)^.

A crise sanitária advinda da pandemia de COVID-19 repercutiu na fragilização dos serviços obstétricos mediante a necessidade de contenção da transmissão viral. Foram incorporadas, nas práticas de assistência ao parto e puerpério imediato, medidas emergentes relacionadas ao plano de contingência que estava sendo implementado no país. Porém, isso ocorreu em detrimento das boas práticas obstétricas no que diz respeito à igualdade de acesso a cuidados perinatais de qualidade e à proteção de direitos previstos na legislação vigente^(17)^. Assim, o contexto pandêmico acarretou à população situações de vulnerabilidade nas dimensões individuais e coletivas, agravando e revelando desigualdades^(22,23)^. Destaca-se o acesso aos serviços de saúde como direito fundamental de segunda geração, constituindo-se a ausência ou insuficiência de acesso a diagnóstico, tratamento, insumos. Destacam-se, também, aspectos como distância geográfica, filas de espera, falta de leitos, entre outros, nos quais são vivenciados processos de vulnerabilização^(13)^.

Somado a isso, as medidas restritivas impostas pela maior parcela de instituições de saúde foram responsáveis pela mudança temporária de algumas práticas como: proibição ou controle do(s) acompanhante(s) durante o trabalho de parto, parto e pós-parto; restrição absoluta de visitantes; recurso a intervenções obstétricas desnecessárias; critérios para contato pele a pele; clampeamento do cordão umbilical e alojamento conjunto; e cuidados na amamentação^(10,24)^.

Nos discursos dos entrevistados, é possível perceber que as medidas restritivas, em virtude da contenção viral, impossibilitaram, para alguns, a participação mais efetiva no período parturitivo e puerperal. Essa realidade também se assemelha ao cenário internacional, quando se evidenciou que companheiros e pessoas de apoio às mulheres foram impactados negativamente pelas restrições nos serviços de maternidade durante a pandemia, onde vivenciaram sentimentos de isolamento, sofrimento psicológico e redução do tempo de vínculo com os bebês^(25)^. Tal fato demanda preocupação, visto que o envolvimento do pai nesse momento é pertinente, na medida em que acarreta efeitos positivos na evolução do trabalho de parto, na recuperação puerperal e na fonte de apoio emocional^(26)^. Mesmo entendendo a importância e necessidade dessas medidas, compreende-se que os impactos gerados por fatores que criam vulnerabilidades podem ser mitigados por práticas que garantam a humanização no atendimento, mesmo em tempos de crise sanitária como a causada pela COVID-19^(27)^.

Observou-se neste estudo que as medidas restritivas nas maternidades durante o período pandêmico não foram implementadas de maneira uniforme, mas sofreram flexibilizações de acordo com a política de cada instituição, de modo que alguns hospitais mantiveram, ainda que parcialmente, a efetivação da Lei do Acompanhante^(4)^ nas maternidades em estudo. A lei foi implantada em nível nacional há cerca de 19 anos, e ainda assim verifica-se que as mulheres não conseguem usufruir totalmente dos direitos assegurados pela lei vigente, o que caracteriza um ato de violação dos direitos reprodutivos do casal por parte dos Serviços de Atenção à Saúde da Mulher^(28)^, gerando uma condição vulnerabilizante ao pai, à sua parceira e ao recém-nascido.

Desse modo, acredita-se ser fundamental a presença do acompanhante no trabalho de parto, parto e pós-parto, incluindo pessoas confiáveis e profissionais capacitados. É preciso reconhecer o acompanhante como um importante ator social nesse momento, uma vez que reflete sobre o processo fisiológico do parto e fortalecimento de vínculos^(2)^.

Percebeu-se que os discursos remetem à diversidade de sentimentos e emoções, tanto positivos quanto negativos, que envolveram a presença paterna durante o período parturitivo-puerperal. Sentiam-se felizes por estarem presentes, mas também eram acometidos pelo sentimento de insegurança e medo da transmissão do vírus. Dessa forma, entende-se que a gestação é marcada por um período de instabilidade emocional, e a pandemia, de modo geral, desencadeou níveis de depressão e ansiedade em gestantes, indicando uma maior necessidade de suporte familiar e uma qualificação da assistência prestada pelos profissionais de saúde.

Nesse sentido, as entrevistas destacaram o impacto na situação psicoemocional da figura paterna, incluindo o medo e a preocupação de contaminar a parceira e o filho, desencadeando repercussões negativas sobre a participação efetiva do pai, vulnerabilizando-o. De acordo com o modelo de VS, a situação psicoemocional diz respeito a uma condição de experiência subjetiva em um contexto específico que envolve desde crenças, sentimentos, bem-estar, percepções, desejos, valores até a saúde mental^(14)^.

Estudo encontrou resultados semelhantes, ao identificar a preocupação de pais relacionada a um possível contágio dentro da unidade familiar e, portanto, o surgimento de uma dualidade de sentimentos: se, por um lado, há a alegria da chegada do filho em meio a um contexto caótico, por outro, a compreensão da existência do risco de transmissão associado à presença do companheiro junto à mulher dá lugar a um sentimento de culpa e tensão^(29)^. Tais informações são indicativas de um cenário inquietante, ao passo que o medo de ser infectado com a COVID-19 aumenta os sintomas de depressão e ansiedade, e adversidades entre os cônjuges^(24,30)^.

Corroborando esses achados, constatou-se que a pandemia pode causar alguns transtornos mentais nos homens, como a depressão pós-parto paterna^(31)^. Desse modo, faz-se importante a participação das autoridades e profissionais de saúde no que tange às necessidades desse grupo, mediante o desenvolvimento de ações que englobam triagem, diagnóstico, prevenção e tratamento. Enfatiza-se, também, que a depressão em mães tem sido estudada durante o cenário pandêmico, evidenciando-se na literatura científica que as mulheres têm sido mais suscetíveis a transtornos mentais, porém a saúde mental dos pais ainda é subestimada^(32)^.

Com base nisso, observa-se que a pandemia de COVID-19 impôs ao pai condições de VS, considerando-se urgente o desenvolvimento de um modelo de cuidado em saúde pública que engloba ações de promoção, prevenção e intervenção, no âmbito familiar, dentro dos territórios ou das comunidades, com o objetivo de promover maior participação paterna em contexto adverso. Os profissionais de saúde devem estimular a criação de espaços de apoio às famílias em situação de vulnerabilidade, garantindo autonomia, estabelecendo vínculos de confiança e formando agentes de mudança para melhoria da assistência^(33)^, e melhor enfrentamento dos desafios em tempos de crise sanitária^(27)^.

Ademais, as falas paternas durante o puerpério imediato remetem ao desempenho dos mesmos nos cuidados com a cônjuge e o filho, contribuindo para o fortalecimento de vínculos e a construção efetiva da figura paterna. Entretanto, os resultados revelaram pais que auxiliaram nas demandas cuidativas, mas também pais que expressaram insegurança e enfrentaram desafios diante dessa realidade.

O não envolvimento paterno nas práticas de cuidado durante o período parturitivo-puerperal pode estar associado a um letramento funcional deficiente do pai, o que impede a busca e utilização de conhecimento apreendido na execução de cuidados em saúde para si mesmo ou para gestante e bebê^(34)^. Assim sendo, essa situação se configura como condição de precariedade que potencializa a vulnerabilização desses participantes e prejudica a participação parental efetiva. Concorda-se que o pai sem letramento satisfatório^(35)^ tem sua capacidade de tomada de decisão em saúde afetada. Consequentemente, sua autonomia, diante das demandas de cuidado, também está comprometida.

Ademais, entende-se que as questões culturais, advindas do patriarcalismo e machismo, que reproduzem desigualdades de gênero, são reforçadas ainda hoje na sociedade, sobretudo em regiões do nordeste do país, como no semiárido brasileiro^(36)^, também contribuindo para esse cenário de ausência da “paternagem”. Portanto, persistem os paradigmas acerca da responsabilidade pelo cuidado com os filhos, gerando o não envolvimento paterno desde a gestação. As diferenças entre gêneros refletem na distribuição de atribuições, quando a mãe assume o papel de cuidar e zelar, enquanto o pai se torna responsável por prover os materiais e recursos para a família^(37)^. Ressalta-se que as desigualdades de gênero, o machismo e o desempenho de papeis tradicionais são apontados como elementos potencializadores da VS. Assim, o período parturitivo-puerperal segue marcado pela presença de um pai espectador, não de um participante ativo.

Por esta razão, destacam-se o desconhecimento e a falta de informação sobre os métodos não farmacológicos de alívio da dor no parto relatados por P3, o que elucida a fragilidade da educação em saúde ofertada para os companheiros dessas gestantes, de modo a afetar negativamente o envolvimento dos mesmos durante o processo parturitivo. Tal situação retrata a faceta programática da VS que perpassa esse contexto, caracterizada pela desarticulação dos atores dos serviços de saúde durante a assistência pré-natal^(15)^. A situação programática é entendida neste estudo incluindo elementos da infraestrutura e processo de trabalho, e ainda situações que envolvem dificuldade de acesso e violação dos direitos humanos^(38)^.

Diante da presença dessas situações programáticas na assistência materno-infantil, a VS é intensificada, e a capacidade de agenciamento dos participantes diante dessas condições de precariedade tende a ser prejudicada. Dessa forma, o acompanhamento pré-natal deve ser qualificado de modo a ser implementado com ênfase nas necessidades de saúde da mulher, fortalecendo a cultura do acompanhante desde as consultas no nível primário, a fim de ofertar informações sobre o parto e o pós-parto e contribuir para mitigação dessas VSs^(39)^.

Nesse ínterim, é fundamental a realização efetiva de uma educação em saúde paterna para estimular a participação do pai desde o período gravídico até o puerpério, não apenas na perspectiva de provisão material, mas, sobretudo, no envolvimento emocional com a gestante, com o bebê e no incentivo da relação de parentalidade^(19)^. Acredita-se que os pais conscientes quanto ao seu papel no período do parto e pós-parto tendem a agenciar suas VSs para assumir uma participação efetiva, mesmo em períodos de crise.

A experimentação do nascimento, não como espectador, mas como participante direto, ressignifica a identidade masculina a partir da reconstrução do ideário da paternidade. Acredita-se, então, que a exclusão do parceiro nos cuidados de maternidade nas condições pandêmicas teve impactos negativos no vínculo familiar^(39-41)^.

Logo, compreende-se que, ao partir do pressuposto que as VSs emergem das relações de poder entre o sujeito e o social, o aspecto relacional da participação paterna no período parturitivo-puerperal precisa ser considerado. Não se deve apenas destacar a presença paterna do ponto de vista físico para atender à Lei do Acompanhante, mas, na consulta pré-natal, a participação e o envolvimento do pai com a mãe e o bebê devem ser abordados de forma relacional e dinâmica, para que as condições precárias possam ser gerenciadas e as vulnerabilidades mitigadas^(13)^.

Do contrário, ao tratar o pai como um ser estático que apenas acompanha observando o processo, as vulnerabilidades são intensificadas, ao passo que não podem ser transformadas por um sujeito que não assume uma identidade performativa, capaz de ressignificar experiências, discursos, vivências e práticas sociais.

### Limitações do estudo

A não abordagem de outros atores envolvidos no período parturitivo-puerperal, o que ampliaria o olhar para o objeto de estudo e os resultados durante o período de pandemia, pode não refletir as experiências em tempos não pandêmicos, o que pode limitar a generalização dos resultados.

### Contribuições para área da enfermagem

Os resultados contribuem para reflexões acerca das VSs que envolvem a experiência paterna durante o período parturitivo-puerperal de sua companheira que foram intensificadas durante a pandemia de COVID-19. Percebe-se que a atuação do enfermeiro na realização do pré-natal do parceiro contribui para o empoderamento dos participantes, com vistas ao agenciamento de suas vulnerabilidades, por meio de educação em saúde eficaz e acompanhamento qualificado. Ademais, considerando a forte influência da enfermagem nesse cenário, os dados do presente estudo também apontam para a necessidade de fomentar intervenções que favoreçam a inclusão da figura paterna na rotina de cuidados com a companheira e recém-nascido, mesmo em tempos não pandêmicos ou outros possíveis contextos pandêmicos.

## CONSIDERAÇÕES FINAIS

O estudo revelou que as experiências paternas no período parturitivo-puerperal foram marcadas por uma participação que, embora não tenha sido amplamente restringida durante a pandemia de COVID-19, ainda enfrenta diversos desafios para se efetivar plenamente. Apesar de os dados desta pesquisa terem sido coletados no contexto pandêmico, eles apontam para uma problemática atemporal: o envolvimento paterno nas demandas de cuidado materno-infantil. As vulnerabilidades associadas a esse contexto específico precisam ser reconhecidas e mitigadas.

A educação em saúde voltada à paternagem necessita ser implementada nas consultas de pré-natal, haja vista o preparo da gestante e do seu companheiro para vivenciar o período parturitivo-puerperal de forma segura e efetiva. É necessário reorientar o serviço de atenção primária no contexto pós-pandêmico, preparando-o, inclusive, para o enfrentamento de futuras crises. Para tanto, urge o desenvolvimento de uma assistência obstétrica humanizada, direcionada não apenas para o desfecho adequado do nascimento, mas para os processos relacionais que perpassam esse contexto e influenciam experiências positivas no que diz respeito ao envolvimento paterno desde o período gravídico, à experimentação do parto e nascimento, não como espectador, mas como protagonista no exercício de sua paternidade.

Sugere-se a realização de outras pesquisas comparando a realidade entre o serviço público e privado, assim como estudos que se detenham em avaliar os efeitos da participação paterna nos desfechos obstétricos, visto que o objetivo desta pesquisa não é esgotar a compreensão da temática, mas abrir novas possibilidades de estudos.

## Data Availability

Os dados de pesquisa estão disponíveis no corpo do artigo.
